# Anxiolytic properties of A_1_ adenosine receptor PAMs

**DOI:** 10.18632/oncotarget.13802

**Published:** 2016-12-05

**Authors:** Fabrizio Vincenzi, Pier Andrea Borea, Katia Varani

**Affiliations:** Department of Medical Sciences, Pharmacology Section, University of Ferrara, Ferrara, Italy

**Keywords:** adenosine, anxiety, positive allosteric modulation, benzodiazepines, A1 receptors

Anxiety and anxiety disorders are among the most prevalent mental health conditions worldwide. Despite the high prevalence rates, anxiety is often an underestimated and undertreated clinical problem. Current treatment strategy for the management of anxiety mainly involves the use of selective serotonin reuptake inhibitors and benzodiazepines. Although widely prescribed and effective in reducing acute anxiety, benzodiazepines are associated with different adverse effects such as impaired cognition and coordination, physiological and psychological dependence, sedation, and memory loss [1]. Furthermore, they have a potential risk of overdose/intoxication when mixed with other central nervous system (CNS) depressant such as alcohol or opioids. For these reasons, safer and fast-acting therapeutic alternatives are highly desirable.

Various studies suggested the involvement of adenosine in the regulation of anxious states. Adenosine is a ubiquitous nucleoside that acts as a neuromodulator in the CNS, controlling neuronal excitability and modulating neurotransmitter release [2]. The action of adenosine is mediated by four G-protein coupled receptors named as A_1_, A_2_A, A_2_B and A_3_ subtypes. Several works have identified A_1_ adenosine receptors (ARs) as potential target for the development of novel strategies in the management of anxiety. In particular, enhanced anxiety has been observed in A_1_ARs knockout mice. Interestingly, electrophysiological recordings from hippocampal slices revealed that adenosine-mediated inhibition of excitatory glutamatergic neurotransmission were abolished in A_1_ARs knockout mice [3]. Previous work have demonstrated the anxiolytic-like effect of A_1_AR activation by means of the well-known agonist CCPA [4]. Despite their promising therapeutic potential, the use of A_1_AR agonists has been hampered by numerous side effects, poor receptor subtype selectivity and receptor desensitization.

To overcome the problems associated with the utilization of A_1_AR agonists, we have developed a series of positive allosteric modulators (PAMs) as a potential alternative for A_1_ARs-targeted therapies [5]. Allosteric receptor modulation represents an attractive concept in drug targeting offering remarkable potential advantages over conventional agonists. In particular, since PAMs enhance the effect of endogenous agonists, they are expected to have a much lower side effect potential than orthosteric agonists, a low propensity for receptor desensitization and a high selectivity for a given receptor subtype. TRR469 is one of the most potent PAMs for A_1_ARs so far synthetized. In a previous work, we have demonstrated the anti-nociceptive effects of TRR469 in two models of acute pain such as writhing and formalin tests and in chronic streptozotocin-induced diabetic neuropathy with effects comparable to those of the reference analgesic morphine. In contrast to the A_1_AR agonist CCPA, TRR469 did not displayed locomotor or cataleptic side effects [6].

From these encouraging results, in a subsequent work we have explored the potential anxiolytic-like activity of TRR469 in mice [7]. In two classical behavioral tests such as the elevated plus maze and the dark/light box test, TRR469 exhibited robust anxiolytic-like effects comparable to those of benzodiazepine diazepam, which was used as a positive control. Pretreatment with the A_1_AR antagonist DPCPX abolished the anxiolytic-like effect of TRR469 demonstrating the involvement of this receptor subtype. In the open field test, TRR469 increased the time spent in the center without affecting the locomotor activity of the mice while in the marble-burying test it reduced the number of marbles buried confirming its anxiolytic-like properties. Anxiety disorders have high rates of comorbidity with alcohol and drug abuse. Even if benzodiazepines are generally considered safe, their interaction with ethanol is a major concern as it produces a marked impairment of psychomotor performance. In contrast to diazepam, we have shown that TRR469 did not enhance ethanol-induced loss of righting reflex suggesting the lack of sedative side effects and interaction with ethanol. Moreover, TRR469 did not impair rotarod performance either *per se*, or in the presence of ethanol while diazepam produced motor coordination impairments greatly potentiating the effect of ethanol. In brain regions intimately involved in stress, anxiety and emotional responses, TRR469 significantly increased the number of A_1_ARs recognizable by the agonist radioligand [^3^H]- CCPA. In membranes obtained from the same tissues, we have shown that TRR469 was able to greatly increase the affinity of the adenosine analogue CCPA [7]. This result is particularly important because one of the great advantages of PAMs is their ability to increase endogenous agonist affinity, enhancing the activation of the receptor in a more physiological way.

Taken together, our findings further corroborate the therapeutic potential of PAMs and in particular those acting on A1ARs such as TRR469 which has shown excellent anti-nociceptive and anxiolytic-like properties in preclinical studies. The affinity increase induced by TRR469 suggest the possibility to exploit the effect of endogenous adenosine in a more physiological way than using an exogenous orthosteric agonist. Given the extensive role of adenosine as a neuromodulator and its involvement in various pathological conditions, further exciting therapeutic properties of A_1_AR PAMs could be discovered.
